# Using Patient Satisfaction Scores to Compare the Performance of Nurse Practitioners As Compared to Doctors in Direct Endoscopy Clinics: A Novel Pilot Trial

**DOI:** 10.7759/cureus.80502

**Published:** 2025-03-13

**Authors:** Aathavan Shanmuga Anandan, David Huynh, Peter Hendy

**Affiliations:** 1 School of Medicine, The University of Queensland, St. Lucia, AUS; 2 Gastroenterology and Hepatology, Mater Hospital, South Brisbane, AUS

**Keywords:** gastroenterology and endoscopy, nurse-led clinic, nurse practitioner (np), outpatient clinic, quality improvement research

## Abstract

The rising aging demographic alongside an expansion of the bowel cancer screening program within Australia has resulted in growing endoscopy wait lists. Compounding this with physician shortages nationwide has prompted health systems to expand the role of nurse practitioners (NPs) into traditionally physician-led settings. This pilot trial explored patient satisfaction with NP-led care compared to physician-led care in direct access endoscopy (DAE) clinics. Patients attending DAE clinics were surveyed regarding their experiences before and after their consultation. The survey assessed professionalism, communication, and overall satisfaction using a 5-point Likert scale. Among the surveyed 71 participants, patients who saw NPs rated their experiences more positively in terms of professionalism and friendliness, although satisfaction levels were comparable overall between NPs and physicians. Despite preconceived biases favouring physicians, 90.3% of patients indicated they would be willing to see an NP again. These findings suggest that patient satisfaction with an NP-led DAE clinic parallels that of a physician-based model. The study also highlights the importance of addressing patient misconceptions about NPs to foster greater acceptance of NP-led care. This proposed NP-led model of care would allow gastroenterologists to maximise their procedural endoscopy time by minimising time spent in the outpatient clinic. Future research with larger, multi-site studies could further validate these results and assess clinical outcomes to support the broader implementation of NP-led DAE clinics.

## Introduction

Physician shortages have become increasingly prevalent in healthcare systems worldwide, prompting a shift toward expanding the roles of nurse practitioners (NPs) to address patient care delivery [1,2]. The integration of NPs into specialised clinical settings has raised important questions about the comparability of care to physicians [3,4]. Expansion of the NP role into novel healthcare settings requires assessing both outcomes and patient satisfaction as a metric for measuring quality of care [5].

NPs have been deployed across various healthcare settings, from primary care to emergency departments with positive patient feedback [3,4]. Patient satisfaction encompasses key dimensions such as interpersonal interactions, perceived clinical competence, communication effectiveness, and overall quality of care [6,7]. One study compared NPs and respiratory physicians in specialised bronchiectasis clinics, noting no significant differences in health outcomes, and numerically greater quality-of-life outcomes post-NP review [8]. This proved consistent with a similar study comparing rheumatology NPs with junior doctors’ clinics where clinical outcomes were comparable, with increased overall satisfaction following NP review [9]. These findings have been replicated in Australian hospital departments where clinical outcomes between care providers are similar, yet NPs consistently provide better health education to patients and follow-up instructions [10,11]. Despite extensive evidence supporting the outcomes and experiences of patients treated by NPs, inherent patient bias exists in a subsect of the population [12,13]. This bias is driven by a lack of understanding of the clinical expertise and scope of practice of NPs within a subspecialised environment.

Direct access endoscopy (DAE) clinics provide a streamlined, outpatient, hospital-based service to allow patients to receive a timely evaluation of gastrointestinal symptoms and ultimately referral for endoscopy. DAE clinics expedite the care pathway from initial referral to specialist consultation, and ultimately diagnostic intervention with endoscopy where appropriate. Increasing rates of referral to DAE clinics are a net result of a multitude of factors not limited to population growth, a rising aging population, and the expansion of the National Bowel Cancer Screening Program [14,15]. Modelling studies have projected that current approaches to endoscopy would lead to the number of patients waiting longer than clinically recommended doubling within five years [15]. NP-led DAE clinics allow specialist gastroenterologists to reappropriate time to endoscopy services, reducing the anticipated increase in endoscopy wait times.

In response to the growing burden, a pilot trial of NPs in DAE clinics was created to optimise patient outcomes and allow prompt evaluation and endoscopy referral. By utilising patient-reported experiences, the project had two objectives: to understand patient expectations and acceptance of a newly introduced NP-led DAE clinic at Mater Hospital Brisbane (MHB), and assess the impact of inherent patient bias on patient satisfaction of NPs versus physicians. These objectives would aid in guiding clinical decision-making and long-term patient outcomes.

## Materials and methods

Patients attending DAE clinics at MHB were surveyed across two months from March 2024 to May 2024. Patients referred to DAE clinics have been initially triaged by a gastroenterologist who has perceived the patient's presenting symptoms as warranting streamlined access to endoscopy. On arrival, patients were asked by the administration team whether they wished to partake in a quality improvement project with dissemination plans to a journal where all data would be de-identified. There were no minimum criteria for eligibility, except being a patient attending DAE clinics at MHB within the recruited time period. The physicians involved were either advanced gastroenterology trainees or specialist gastroenterologists, and the NP involved was a specialised gastroenterology NP.

Patients completed two survey sections: a pre- and post-consultation questionnaire using a 5-point Likert scale. The pre-consultation questions were set to determine if the patients had preconceived biases by enquiring whether the patients expected to see a physician or NP and whether they understood the role of an NP. Post-consultation questions assessed three key domains: ‘professionalism and friendliness’, ‘understanding of the procedure’, and ‘communication of the procedure’, alongside a final rating of ‘overall experience’. Patients were also asked if they would have preferred to see a physician or NP following the consult.

Patients were blinded to whether they would see an NP or physician for their endoscopic consultation. Similarly, the healthcare provider was unaware of the patient’s presenting symptoms, negating the possibility of selection bias. Patients were seen based on the earliest availability of healthcare providers and were indiscriminately assigned to either an NP or a physician. All questionnaires were completed anonymously and placed in collection boxes at the outpatient clinic administration station. Ethics approval was not required, as it was deemed a quality assurance project as per local guidelines. This study involved human subjects and informed patient consent was obtained. Data was deidentified to ensure participant privacy and confidentiality.

Data was analysed using Stata Version 18 (StataCorp. 2023. Stata Statistical Software: Release 18. College Station, TX: StataCorp LLC). Results were reported as mean values with confidence intervals (CIs) as determined by patient-reported outcomes using a 5-point Likert score. P-values were determined through Wilcoxon rank sum testing with a two-sided p-value of 0.05 used to establish significance.

## Results

**Figure 1:**
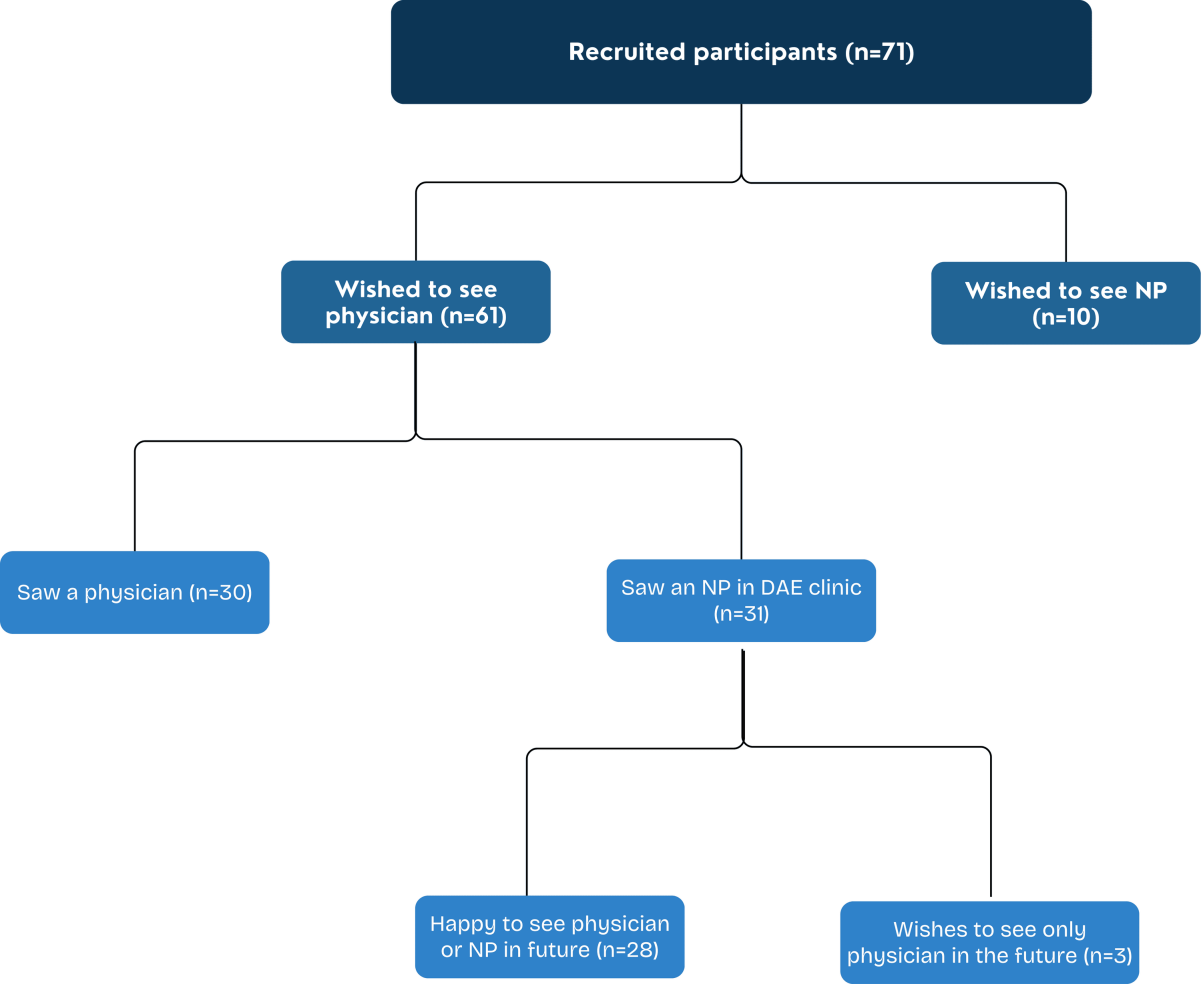
Schematic representation of patient flow in relation to inherent bias observed in a DAE clinic preceding and following either physician or NP review DAE = Direct Access Endoscopy, NP = Nurse Practitioner

Across the examined period, 92 patients offered to participate in the questionnaire, with 71 (77%) patients completing questionnaires. Thirty-three (46%) patients saw a doctor, and 38 (54%) saw an NP (Figure 1).

NP ratings were significantly greater than physicians in the domain of ‘professionalism and friendliness’ (physician 4.2 (95% CI 3.8 - 4.7), NP 4.7 (95% CI 4.5 - 4.9), p = 0.03) (Table 1). Similarly, NPs scored numerically greater across ‘understanding of the procedure’ (physician 4.5 (95% CI 4.3 - 4.7), NP 4.7 (95% CI 4.5 - 4.8), p = 0.21), ‘communication of the procedure’ (physician 4.5 (95% CI 4.3 - 4.7), NP 4.6 (95% CI 4.5 - 4.6), p = 0.4), and ‘overall experience’ (physician 4.0 (95% CI 3.5 - 4.5), NP 4.3 (95% CI 3.9 - 4.7), p = 0.29)) (Table 1).

**Table 1:** Mean (95% CIs) score of patient-reported experiences between doctor (n=33) and NP (n=38) review in a DAE clinic using a 5-point Likert scale CIs = Confidence Intervals, NP = Nurse Practitioner, DAE = Direct Access Endoscopy. *P-value threshold of <0.05 was considered significant.

Outcome	Wished to see a physician, seen by a physician	Wished to see a physician, seen by an NP	P-value
Overall experience	4.0 (95% CI 3.5–4.5)	4.3 (95% CI 3.9–4.7)	P = 0.29
Professionalism and friendliness	4.2 (95% CI 3.8–4.7)	4.7 (95% CI 4.5–4.9)	P = 0.03*
Understanding of the procedure	4.5 (95% CI 4.3–4.7)	4.5 (95% CI 4.5–4.9)	P = 0.65
Communication of the procedure	4.5 (95% CI 4.3–4.7)	4.5 (95% CI 4.4–4.8)	P = 0.72

Of the total 71 patients, 61 preferred to be seen by a physician, and 10 by an NP. Of these 61 patients, 51% (n=31) were reviewed in a DAE clinic by an NP, with the remainder 49% (n=30) being seen by a physician (Table 2).

**Table 2:** Mean (95% CIs) scores reported in Group A (n=33) compared to Group B when seen in a DAE clinic using a 5-point Likert scale Group A represents patients who wished to be seen by a physician and ultimately were reviewed by a physician. Group B represents patients who wished to be seen by a physician and ultimately were reviewed by an NP. CIs = Confidence Intervals, DAE = Direct Access Endoscopy, NP = Nurse Practitioner. *P-value threshold of <0.05 was considered significant.

Outcome	Wished to see a physician, Seen by a physician	Wished to see a physician, seen by an NP	P-value
Overall experience	4.0 (95% CI 3.5–4.5)	4.3 (95% CI 3.9–4.7)	P = 0.29
Professionalism and friendliness	4.2 (95% CI 3.8–4.7)	4.7 (95% CI 4.5–4.9)	P = 0.03*
Understanding of the procedure	4.5 (95% CI 4.3–4.7)	4.5 (95% CI 4.5–4.9)	P = 0.65
Communication of the procedure	4.5 (95% CI 4.3–4.7)	4.5 (95% CI 4.4–4.8)	P = 0.72

Of these 31 patients, 28 expressed no reservations about seeing either an NP or a doctor following the consultation. The average scores for patients who wished to see a physician and ultimately were seen by a physician (Group A, n=30) rated their ‘overall experience’ 4.0 (95% CI (3.5-4.5)), ‘professionalism and friendliness’ 4.2 (95% CI (3.8-4.7)), ‘understanding of the procedure’ 4.5 (95% CI (4.3-4.7), and ‘communication of the procedure’ 4.5 (95% CI (4.3-4.7)). Conversely, patients with preconceived biases who wished to see a physician and were ultimately reviewed by an NP (Group B, n=31) rated their ‘overall experience’ 4.3 (95% CI (3.9-4.7)), ‘professionalism and friendliness’ 4.7 (95% CI (4.5-4.9)), ‘understanding of the procedure’ 4.5 (95% CI (4.5-4.6)), and ‘communication of the procedure’ 4.5 (95% CI (4.4-4.8)).

## Discussion

The overall comparable rates of patient-reported satisfaction between NPs and physicians support the acceptability of the novel pilot trial in DAE clinics. These findings proved consistent with existing NP utilisation in a breadth of various clinical settings from primary care to emergency departments. Patient satisfaction across all assessed domains proved greater for NPs compared with physicians, underscoring the potential of NPs to play a significant role in delivering high-quality care in specialised clinical settings. Broader adoption of this model would allow gastroenterologists to maximise procedural endoscopy time and mitigate growing endoscopy wait lists nationally.

The findings reflect the comprehensive specialised training NPs undertake to provide holistic care in their respective field. Their ability to foster strong patient-provider relationships can enhance patient perceptions of care quality [16]. Moreover, given that NPs often spend more time with patients during consultations, allowing for thorough discussions of treatment options and addressing patient concerns, may contribute to improved satisfaction rates [17]. These findings indicate that patient acceptability of NP involvement in DAE clinics is high, suggesting this is not a barrier to ongoing implementation. Future research observing whether clinical outcomes are similar, as assessed by comparing decision-making between NPs and physicians, could provide objective data regarding the safety of future expansion of the pilot trial.

Rising endoscopy-related health expenditure per capita in Australia results from population growth, increasing median ages, and bowel cancer screening expansion, leading to a strain on the health budget [18]. The utilisation of NPs in settings such as DAE clinics benefits health services by optimising economic resources through the employment of an alternative, lower-cost NP model of care [19-21]. Moreover, allowing physicians to reprioritise focus from DAE clinics to performing endoscopy would allow for a reduction in endoscopy wait times. Numerous case studies have explored the cost feasibility of NP-led models of care in various appropriate healthcare settings, with a consensus that care quality parallels that of physicians at reduced cost [19-21].

This pilot trial also explored whether preconceived perceptions of NPs influenced how patients rated their quality of care. The results support the notion that inherent bias against NPs failed to influence patients’ rating of care, as reflected by comparable rates of satisfaction and 90.3% of patients willing to be seen by an NP in the future following their consultation. This suggests that positive first-hand experiences through consultation overcome pre-existing perceptions. Emphasis on improving patient understanding regarding the scope and skillset of NPs would instil patient confidence and overcome skewed impressions.

This study had three key limitations, which reduced its internal and external validity. First, the study involved the participation of a single NP versus several physicians across the study period. Thus, positive patient ratings of the NP patient cohort may not necessarily be reflective of NPs but rather be influenced by a single person’s positive attributes. These findings could be further validated through the involvement of multiple NPs to standardise the findings across a group of healthcare providers. Second, the study was a single-site study at a tertiary metropolitan hospital. Tertiary institutional experience and patient demographics may not reflect that of smaller hospitals and regional locations. Given the positive patient perceptions of this pilot trial, an expansion of the study to additional hospitals would increase the external validity of the findings. A multi-site study would improve external validity by allowing for broader generalisability, mitigating site-specific bias and implementation variation, as well as surveying a more globally representative diverse population. Lastly, study design limitations associated with the small survey timeframe and sample size surveyed may not be sufficient to detect small but potentially significant differences. Increasing sample size would improve statistical power by allowing for increased effect size detection, reducing standard error, lowering Type II error, and improving precision. Future investigations would increase the sample size surveyed and would provide more robust findings.

## Conclusions

The preliminary results from this pilot trial provide compelling evidence supporting patient acceptability of nurse practitioners (NPs) in direct access endoscopy (DAE) clinics. Patient satisfaction with NPs is comparable to that of physicians, with numerically greater reported professionalism and friendliness. Furthermore, inherent biases against NPs were overcome as demonstrated by the strong willingness for future NP consultations. By alleviating the clinician burden in DAE clinics, a greater focus for physicians can be placed on performing procedural endoscopy, alleviating the growing burden on public hospital endoscopy services. Future research involving larger sample sizes, multi-site involvement, and multiple NPs can further validate the preliminary findings and cement this novel public health approach.
